# EMG-Normalised Kinase Activation during Exercise Is Higher in Human Gastrocnemius Compared to Soleus Muscle

**DOI:** 10.1371/journal.pone.0031054

**Published:** 2012-02-08

**Authors:** Thomas E. Jensen, Robin Leutert, Søren T. Rasmussen, Joshua R. Mouatt, Mette L. B. Christiansen, Bente R. Jensen, Erik A. Richter

**Affiliations:** 1 Molecular Physiology Group, Department of Exercise and Sport Sciences, University of Copenhagen, Copenhagen, Denmark; 2 Motor Control and Biomechanics Group, Department of Exercise and Sport Sciences University of Copenhagen, Copenhagen, Denmark; University of Las Palmas de Gran Canaria, Spain

## Abstract

In mice, certain proteins show a highly confined expression in specific muscle groups. Also, resting and exercise/contraction-induced phosphorylation responses are higher in rat skeletal muscle with low mitochondrial content compared to muscles with high mitochondrial content, possibly related to differential reactive oxygen species (ROS)-scavenging ability or resting glycogen content. To evaluate these parameters in humans, biopsies from soleus, gastrocnemius and vastus lateralis muscles were taken before and after a 45 min inclined (15%) walking exercise bout at 69% VO2_max_ aimed at simultaneously activating soleus and gastrocnemius in a comparable dynamic work-pattern. Hexokinase II and GLUT4 were 46–59% and 26–38% higher (p<0.05) in soleus compared to the two other muscles. The type I muscle fiber percentage was highest in soleus and lowest in vastus lateralis. No differences were found in protein expression of signalling proteins (AMPK subunits, eEF2, ERK1/2, TBC1D1 and 4), mitochondrial markers (F1 ATPase and COX1) or ROS-handling enzymes (SOD2 and catalase). Gastrocnemius was less active than soleus measured as EMG signal and glycogen use yet gastrocnemius displayed larger increases than soleus in phosphorylation of AMPK Thr172, eEF2 Thr56 and ERK 1/2 Thr202/Tyr204 when normalised to the mean relative EMG-signal. In conclusion, proteins with muscle-group restricted expression in mice do not show this pattern in human lower extremity muscle groups. Nonetheless the phosphorylation-response is greater for a number of kinase signalling pathways in human gastrocnemius than soleus at a given activation-intensity. This may be due to the combined subtle effects of a higher type I muscle fiber content and higher training status in soleus compared to gastrocnemius muscle.

## Introduction

Endurance exercise represents an immense stress to the working muscle due to the increased ATP demand used for ion-pumping and force generation and increased production of potentially harmful by-products such as reactive oxygen species (ROS). To minimise the homeostatic disturbance, the working muscle adapts acutely by a number of mechanisms directed at increasing energy substrate uptake and/or turnover [1]. In addition, repeated exercise bouts elicit changes in gene expression patterns that lead to mitochondrial biogenesis and increased expression of ROS-handling enzymes [1], [2]. Protein phosphorylation is indisputably the most studied mechanism for muscular signal transduction and muscle contraction has been documented to elicit a plethora of protein phophorylation events to elicit adaptations to exercise [3].

Rodent muscles have been used extensively to identify and characterise the ever-expanding kinase signalling networks activated by muscle contraction. Conceptually, muscles are often divided into slow-twitch (type I) and fast-twitch (type II) fibers dictated by their myosin heavy chain (MHC) isoform expression [4]. Human muscle groups are mostly a homogenous mix of slow and fast-twitch fibers whereas rodent muscle groups exhibit a more extreme division into either type I or type II fiber-dominated muscles [5]–[10].

In both rodents and humans, many endurance-training responsive proteins are expressed in an activity-pattern dependent manner, including glucose-handling proteins and mitochondrial markers [1]. However, in mice in particular, there are clearly also a number of proteins with expression confined to certain muscle groups. For instance, the gamma3 subunit of contraction-activated 5′-AMP-activated protein kinase (AMPK) and its downstream substrate protein, TBC1D1, is lowly expressed in mouse slow-twitch oxidative soleus compared to more fast-twitch glycolytic muscles [11]–[14]. Although MHC isoform-distribution is more mixed in humans than rodents, the expression of other potential muscle group-specific proteins may be controlled by distinct transcriptional regulators [4]. It is currently unknown whether any of the muscle group-restricted proteins in mice are enriched in certain muscle-groups in humans.

Recent rat studies have also suggested that both the resting and in situ contraction-induced phosphorylation of a number of kinases correlates inversely with the mitochondrial content of different parts of the tibialis anterior muscle [15]. This might relate to higher ROS-production per mitochondria in less oxidative muscle [16], [17]. To our knowledge, no study has attempted to compare basal and exercise-stimulated kinase phosphorylation in different human muscle-groups at a comparable work-load.

Here, the expression of selected proteins, some of which show muscle group-confined expression in mice, was evaluated in human soleus, lateral gastrocnemius and vastus lateralis-part (VL) of the quadriceps femoris muscle. Furthermore, a human inclined walking exercise model was employed with the aim of comparing resting and exercise-induced kinase responses in human soleus and gastrocnemius muscle at a comparable level of activation.

## Results

**Figure 1:**
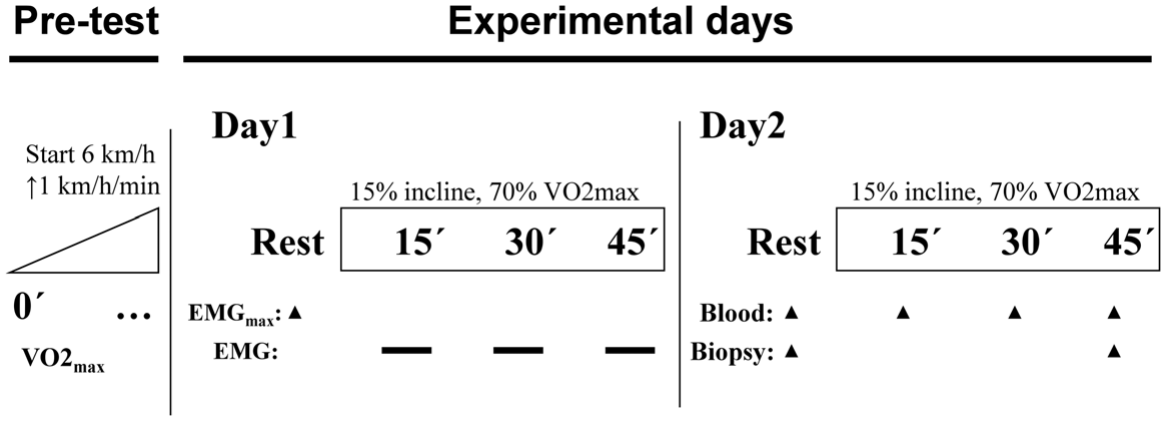
Overview of experimental protocol for pre-test day and experimental days 1 and 2.

A schematic overview of the experimental protocol is shown in figure 1. All subjects completed a 15% inclined 45 min uphill walking bout covering a mean distance of 4770 m and corresponding to a 715 m elevation. This elicited a mean oxygen uptake of 69.1±1.6 %VO2 max and changes in various blood metabolites and hormones typical of whole-body exercise at this intensity including increased lactate and catecholamines and lower insulin (table 1).

**Table 1:** Blood parameters measured as indicated at various time-points during an inclined walking exercise-bout at ∼70% VO2max.

Parameter	Rest	15 min.	30 min.	45 min.
Glucose (mM)	5.13±0.19	5.65±0.18	6.03±0.34*	6.35±0.43*
Lactate (mM)	0.93±0.1	3.52±0.49*	3.87±0.38*	3.99±0.56*
Free fatty acids (µM)	558±106	320±40*	379±44	453±68
Insulin (µU/ml)	3.55±0.54	2.76±0.52	1.77±0.24**	1.47±0.24***
Epinephrine (nM)	0.76±0.07	1.61±0.16	2.07±0.21	2.36±0.24
Norepinephrine (nM)	3.14±0.8	16.36±3.16	21.34±2.25	20.72±3.14

n = 10.

*/**/***p<0.05/0.01/0.001 vs. corresponding resting value.

**Figure 2:**
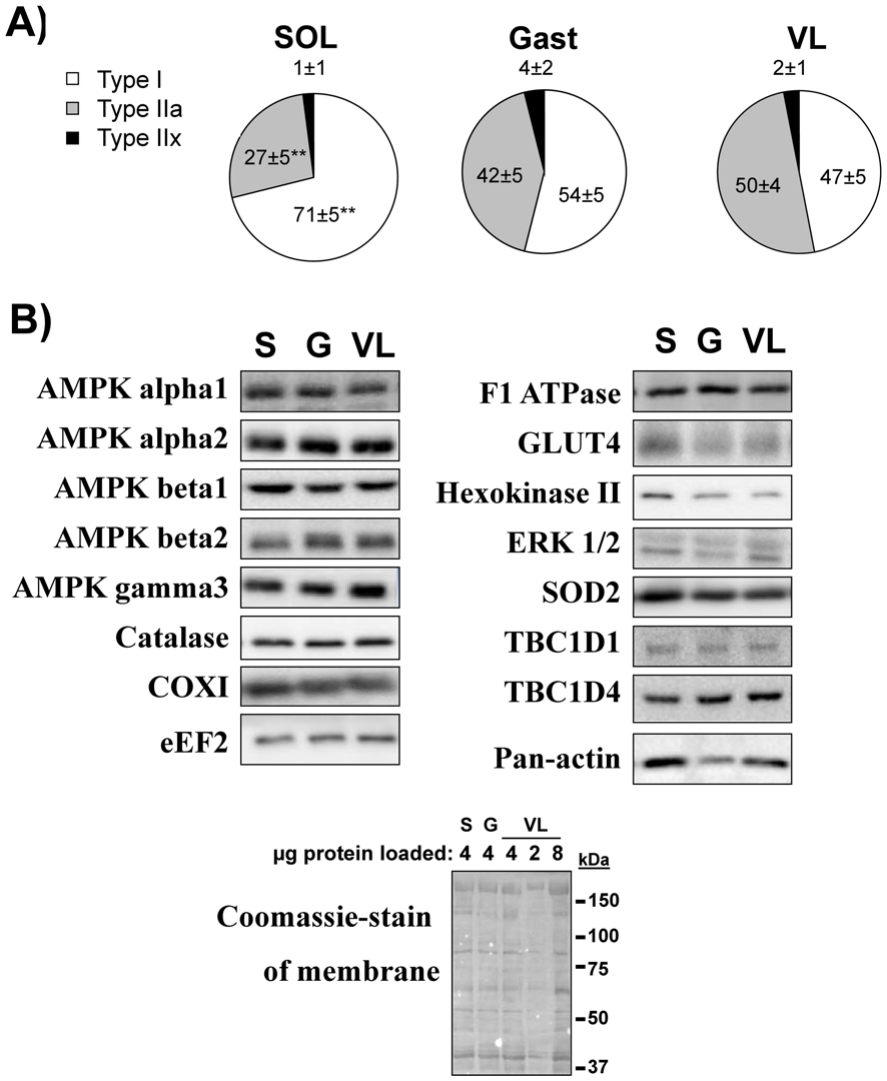
Characterisation of protein expression in different human muscle groups. A) Mean fibertype distribution and B) representative blots of relative protein expression in human soleus (S), lateral gastrocnemius (G) and vastus lateralis (VL) as indicated. ** p<0.01 fibertype-difference soleus vs. VL. n = 7–8 for fibertype-determinations and n = 10 for western blotting.

The measured fibertype-distribution in the biopsies obtained from human soleus, gastrocnemius and VL showed a roughly 20% higher mean type I fiber content and equally lower type IIa fiber content in soleus compared to gastrocnemius and VL (figure 2A). Figure 2B and table 2 also show representative blots and lists the total protein measurements performed on the current biopsy-material. HXII content was higher in soleus compared to gastrocnemius (p<0.01) and VL (p<0.001) and GLUT4 content higher in soleus compared to gastrocnemius (p<0.05). In addition, a strong trend towards a differential expression of the mitochondrial superoxide-converting enzyme, SOD2 was found (p = 0.07 by 1-way ANOVA). All remaining proteins measured, including signalling proteins (AMPK subunits, ERK, eEF2, TBC1D1, TBC1D4), mitochondrial markers (F1 ATPase, COXI) and the hydrogen peroxide-inactivating Catalase-enzyme showed no significant differences in relative expression between muscles. A much used loading control, actin, tended to be higher in soleus compared to gastrocnemius (mean 28% lower) and VL (mean 7% lower). Given that the quantifications for multiple proteins in these samples and coomassie-staining indicates equal protein loading, this may suggest that actin is a poor loading control for comparison between muscles. Overall, the mouse pattern of high enrichment of certain proteins within specific muscle groups is unlikely to be present in humans.

**Table 2:** Relative protein expression quantified from western blots in human soleus, lateral gastrocnemius (Gastroc.) and lateral vastus of the vastus lateralis muscle (VL). SOD2 showed a borderline significant trend (p = 0.07, 1-way ANOVA).

Protein	Soleus	Gastroc.	VL.
**AMPK alpha1**	1.00±0.04	0.98±0.11	0.96±0.11
**AMPK alpha2**	1.00±0.06	1.00±0.08	1.07±0.08
**AMPK beta1**	1.00±0.15	0.81±0.10	0.94±0.12
**AMPK beta2**	1.00±0.10	1.05±0.14	1.16±0.11
**AMPK gamma3**	1.00±0.13	0.87±0.10	1.17±0.12
**Catalase**	1.00±0.13	1.02±0.20	0.80±0.09
**COXI**	1.00±0.12	0.93±0.09	0.81±0.08
**eEF2**	1.00±0.14	0.95±0.19	1.10±0.21
**ERK1/2**	1.00±0.07	1.15±0.10	1.09±0.05
**F1 ATPase**	1.00±0.07	1.01±0.08	0.89±0.12
**GLUT4**	1.00±0.16	0.62±0.11*	0.74±0.13
**Hexokinase II**	1.00±0.15	0.54±0.08**	0.41±0.07***
**SOD2**	1.00±0.08	0.91±0.08	0.80±0.07
**TBC1D1**	1.00±0.15	0.91±0.18	0.97±0.19
**TBC1D4**	1.00±0.06	1.12±0.07	1.07±0.14
**Beta actin**	1.00±0.14	0.72±0.13	0.93±0.14
**Coomassie**	1.00±0.11	0.99±0.11	1.01±0.10

n = 10.

*/**/***p<0.05/0.01/0.001 vs. soleus.

**Figure 3:**
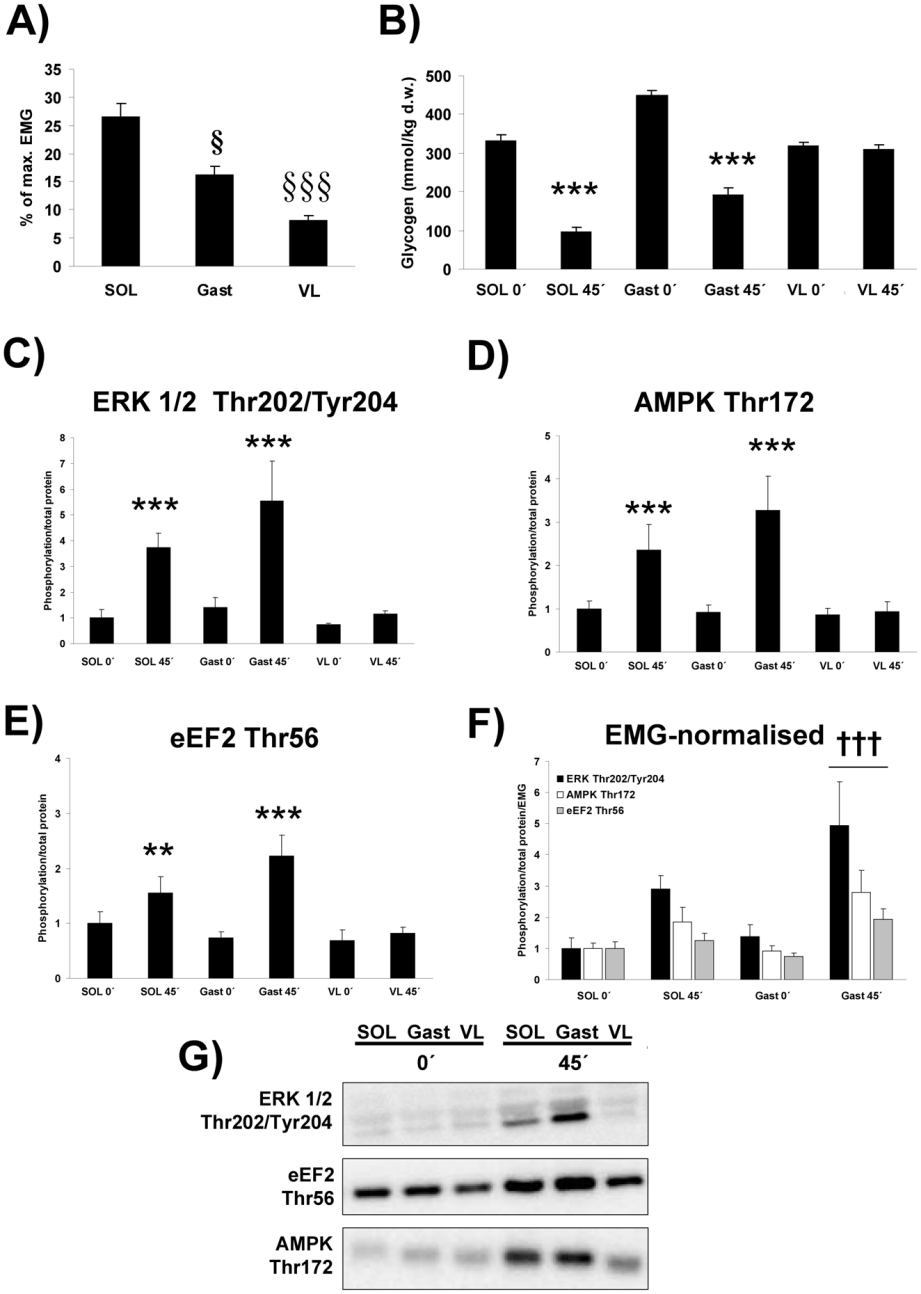
Exercise-induced signalling in different human muscle groups. Relative work-load during exercise-bout evaluated by A) % of maximal EMG and B) glycogen content in human soleus (SOL), lateral gastrocnemius (Gast) and vastus lateralis (VL) before and after inclined walking exercise at ∼70% VO2max. Measured phosphorylations of C) ERK1/2, D) AMPK and E) eEF2 normalised per total protein in SOL, Gast and VL before and after exercise. F) EMG-normalised phosphorylation per total protein in gastroc. vs. SOL. G) Representative blots. §/§§§ p<0.05/0.001 vs. soleus. *** p<0.001 exercise-effect vs. rest within muscle. ††† p<0.001 muscle-effect, n = 10.

Soleus was significantly more active than gastrocnemius measured as both %EMG_max_ (Fig. 3A) and glycogen use (Fig. 3B). Total glycogen tended to be higher in gastrocnemius compared to soleus muscle although this was not significant (Fig. 3B). VL was only minimally activated by the current exercise regimen. Measured as %total glycogen use, the numbers were 70%, 57% and 2% for soleus, gastrocnemius and VL-muscle, respectively.

ERK, AMPK and eEF2 phosphorylation were all significantly higher after exercise in soleus and gastrocnemius, but not in VL (Fig. 3C–E). Interestingly, despite the fact that gastrocnemius was less active than soleus, the absolute mean activation after exercise tended to be greater in gastrocnemius than soleus for all signalling outcomes measured. When normalised to %EMG_max_, gastrocnemius showed a significantly higher general kinase-responsiveness to exercise compared to soleus (Fig. 3F, p<0.001, representative blots in Fig. 3G).

## Discussion

This study examined protein expression and kinase-signalling in 3 different muscle-groups of the lower extremity in humans. Our first aim was to assess whether signalling proteins like AMPK and TBC1D1 and 4, previously found to display muscle-group specific expression in mice, differed between human muscle groups. This was clearly not the case and in general protein expression was found to be similar in different lower extremity human muscles. The second aim was to examine whether resting and exercise-stimulated kinase-signalling differed between human muscles at a given rate of activation as previously shown in rats. Surprisingly, despite comparable expression of mitochondrial marker proteins, glycogen content and only a trend towards lower SOD2 content in gastrocnemius, the latter muscle displayed a ∼60% greater mean exercise-induced increase in phosphorylation-response for the 3 kinase-signalling pathways measured compared to soleus at a given level of EMG-defined muscle activation.

A number of endurance-training responsive proteins have previously been measured in distinct human muscle groups, including GLUT4 and various markers of mitochondrial content [6], [7], [21]–[23]. In general, our results of only subtle differences between muscle groups are in agreement with these studies. This implies that protein expression in human vastus lateralis, the most commonly studied muscle in human exercise-studies, is likely to be generally representative of other muscle groups. The most differentially expressed protein in our study, HXII showed 46–58% higher expression in soleus compared to other muscles. HXII expression is often observed to be very sensitive to endurance-training in humans [24], and the elevated HXII content in soleus is probably reflective of its function as a postural muscle. Of note, some of our total protein measurements (Catalase, COXI, GLUT4, SOD2) show a tendency towards a ∼20% lower level in vastus lateralis compared to the other muscles. Based on the mean standard deviation for these four proteins, a minimum sample size of 17 would be needed to detect differences of this magnitude at the α = 0.05 level by western blotting in future studies. However, even if our sample size is insufficient, the differences between muscle groups for most proteins examined seem to be small (<20%).

During preparation of this manuscript, a study was published measuring selected proteins including TBC1D1 and TBC1D4 in 12 different rat muscles [25]. Similar to humans, TBC1D4 expression was not different and TBC1D1 expression showed a ∼30% difference in expression range between different hindlimb-muscles. This is in stark contrast to the muscle-type confined expression in mice where TBC1D4 is enriched in slow-oxidative muscle and TBC1D1 in fast-twitch glycolytic muscle [12]. Based on this, mice may be unique in their muscle-type restricted expression. Since transgenic mice are a major tool used to chart signalling pathways in muscle-research, this obviously calls for caution when generalising signalling concepts from mice to humans or even other rodent species like rats.

Studies by David Hood's group showed that mitochondrial content was inversely correlated with the resting and in situ contraction-stimulated phosphorylation of a number of kinases in rat tibialis anterior muscle [15], [16]. A putative causal link between kinase phosphorylation and mitochondrial content might be muscle group-specific differences in ROS-mediated signalling. ROS is produced during exercise [2], [26]. In the present study, the lack of difference in SOD2 and catalase-expression did not support that a poorer maintenance of redox-status in gastrocnemius contributed to the higher kinase responsiveness in gastrocnemius compared to soleus.

In rats and humans, low glycogen has been shown to correlate with increased basal phosphorylation and/or exercise or insulin-activation of some kinases including AMPK and Akt [27]–[29]. This occurs by a poorly defined mechanism that does not appear to involve increased skeletal muscle ROS-production in situations with low glycogen in exercising humans [30]. In the present study, the greater exercise-induced kinase-signalling response in gastrocnemius compared to soleus could not be explained by differences in muscle glycogen which tended to be higher in gastrocnemius than soleus muscle.

Endurance-training of rats or humans has been reported to reduce phosphorylations in response to exercise of a number of signalling proteins including AMPK, TBC1D4, mTOR, S6K1, S6 and ERK at the same absolute work-intensity [31]–[35] although the mechanism has not been resolved. Soleus is more posturally active in daily life than gastrocnemius muscle and some of our measured proteins, HXII and GLUT4, would suggest that soleus has, as expected, been more habitually active than gastrocnemius. Thus, it is tempting to ascribe the lower activation-sensitivity in soleus vs. gastrocnemius to a training effect in soleus vs. gastrocnemius in our study. While this phenomenon could relate to an improved maintenance of cellular energy charge for some kinases such as AMPK [34], this does not explain why kinases with other activation-mechanisms would behave similarly. Given that phosphatases can be reversibly inactivated by ROS and affect kinase-activation [36] and the previously discussed link between kinase-activation and mitochondrial ROS, we suspected that reactive oxygen species might be involved. However, our data showing equal mitochondrial enzymes and ROS-handling enzymes in soleus and gastrocnemius clearly do not support this hypothesis. Fascinatingly, endurance-trained athletes have been reported to exhibit a much higher phosphorylation of several proteins including AMPK, S6 and p38 MAPK than resistance trained athletes in response to acute maximal isokinetic leg extension (resistance training) exercise [37]. The opposite is also true in that strength-athletes show a greater kinase-responsiveness than endurance-athletes for some of the same kinases (AMPK, p38 MAPK) after 1 h of moderate-high intensity cycling [37]. Since the greater kinase-responsiveness of endurance-athletes to resistance exercise likely occurs despite higher mitochondrial content and perhaps even a higher percentage of type I fibers with lower intrinsic mitochondrial ROS production as suggested in rat [17], this again appears to be more than a mere function of mitochondrial ROS production. How this regimen-specific attenuation of kinase-responsiveness by training is brought about will be an interesting topic for future studies.

AMPK is an attractive drug target to treat insulin resistance since its activation is likely sufficient to increase glucose uptake in muscle cell culture and mouse muscle [38]–[40] and to drive mitochondrial biogenesis [41]. AMPK is a heterotrimeric αβγ protein-complex and some AMPK activators like AICAR and A-769662 have been shown to require certain AMPK subunits to elicit biological effects [11], [13], [42]. In rodent muscle, AMPK subunit expression differs markedly between muscle groups as does the expression of potential downstream effectors of contraction and insulin-stimulated glucose uptake like TBC1D1 and 4 [11]–[13], [43]. We found near equal expression of these proteins in different human muscle groups, implying that AMPK-dependent signalling mechanisms are likely conserved between most human skeletal muscles.

In conclusion, humans do not exhibit the extreme muscle-specific expression of signalling proteins like AMPK and TBC1D1 and 4 seen in mice. Human gastrocnemius shows a greater phosphorylation response of a number of kinases to exercise at a given degree of EMG-verified activation than soleus. This probably reflects a difference in training-status and muscle fiber type composition between the muscles but occurs despite similar expression of mitochondrial markers, ROS-handling enzymes and glycogen content.

## Methods

### Subjects

10 young moderately trained male subjects [26±2 yr, 184±2 cm, 85±2 kg, 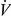 o_2peak_ = 58±2 ml·(kg·min)^−1^] gave their informed written consent to participate in the study, which was approved by the Copenhagen Ethics Committee (reg. no. H-KF277313) and conformed to the Helsinki II Declaration (1996).

### Protocol

#### Pre-test day

The subjects underwent one day of pre-testing and 2 separate experimental days (see Figure 1 for graphic overview). On the pre-test day, anthropometric measurements and maximal oxygen uptake was determined in the fed condition. Upon arrival, the subjects rested in the supine position for 15 min after which resting pulmonary VO2 was determined. The subject then undertook an inclined (15%) treadmill test at increasing speeds to determine VO2_max_ (starting at 6 km/h and increasing 1 km/h every minute until exhaustion) and calculate the experimental walking speed. The 15% incline was shown in a pilot experiment to activate both soleus and gastrocnemius muscle measured as electromyography (EMG) signal during exercise (data not shown).

#### Experimental day 1

On both experimental days the subjects arrived in the morning after an overnight fast. On day 1, the subjects' maximal voluntary EMG was determined for soleus, gastrocnemius and vastus lateralis muscles, and subsequently EMG was recorded with the same surface electrodes during the experimental work-bout (15% incline, walking speed corresponding to 65–70% VO2 max, for 45 min).

Relevant parts of a given subject's legs were shaven, sandpapered and wiped with ethanol after which electrodes were placed in pairs approx. 2 cm. centre–to-centre above soleus, gastrocnemius and vastus lateralis muscles in addition to a reference electrode on the tibia. Maximal EMG responses (highest of 3–5 attempts) were determined for soleus, gastrocnemius and vastus lateralis during a maximal isometric knee extension, a standing isometric loaded heel raise and a sitting isometric heel raise with the knee at a 90° angle. The experimental bout started approx. 90 min after arrival. During the experimental work-bouts, EMG (sampling rate 1000 Hz) was recorded at 5–15 min, 20–30 min and 35–45 min.

#### Experimental day 2

On day 2, a venous catheter was inserted in the forearm antecubital vein for blood sampling. After 60 min of rest, 5 mm incisions were made for each biopsy (1/muscle) above the soleus, lateral head of the gastrocnemius and the vastus lateralis-part of the quadriceps muscles on both legs under local anaesthesia (Xylocaine 2%, Astra-Zenica, Sweden). After an additional 30 min, a resting blood sample and needle biopsy was taken from each of the three muscles in one leg (randomised with respect to dominant leg and biopsy-order), after which the experimental work-bout was begun (15% incline, walking speed corresponding to 65–70% VO2 max, 45 min). Blood samples were collected at 15, 30 and 45 min. After completing the work bout, biopsies were taken from the second non-biopsied leg, and divided for histochemistry and biochemical analyses. The last biopsy was frozen in liquid N_2_ within 3 min after work termination.

### Pulmonary gas analysis

During the incremental maximal walking test, expired air was collected in Douglas bags and analysed as previously described [18].

### EMG analysis

EMG_max_ was calculated as the largest 1 s root mean square value obtained (average of ten 100-ms segments) during the maximum contractions. EMG recorded during the walking exercise was calculated as root mean square amplitudes (100 ms segments) and expressed relative to the maximal EMG response (% EMG_max_). Resting EMG was subtracted from all values. No differences were found between the three recording periods, indicating no development of muscle fatigue. Therefore, data is presented as average of the three periods recorded during the work-bout.

### Blood and plasma analyses

Plasma epinephrine, nor-epinephrine and insulin concentrations were analysed by means of radioimmunoassay kits (High sensitive 2-Cat RIA, Labor Diagnostika Nord GmbH & Co., Germany and Insulin RIA DSL-1600, Diagnostic Systems Laboratories Inc., Webster, TX, USA). The plasma FFA concentration and the blood concentration of glucose and lactate were measured using an automatic analyzer (Hitachi automatic analyzer 912; Boehringer Mannheim, Ingelheim, Germany).

### Muscle fiber typing

Fibertype composition of the different muscles was determined using histochemical ATPase staining of cryosections, as described previously [19].

### Muscle glycogen

Glycogen content was determined as glycosyl units after acid hydrolysis of freeze dried dissected muscle biopsy specimens [19].

### Muscle preparation

All materials were from Sigma-Aldrich (St. Louis, MO, USA) unless stated otherwise. Freeze dried and dissected muscle biopsies were homogenized 1∶15 w/v using a Tissuelyser II (Qiagen, USA) in ice-cold buffer containing 50 mM HEPES (pH 7.5), 150 mM NaCl, 20 mM sodium pyrophosphate, 20 mM β-glycerophosphate, 10 mM NaF, 2 mM sodium orthovanadate, 2 mM EDTA, 1% NP-40, 10% glycerol, 2 mM PMSF, 1 mM MgCl_2_, 1 mM CaCl_2_, 10 µg/ml leupeptin, 10 µg/ml aprotinin, and 3 mM benzamidine. Homogenates were rotated end over end for 30 min at 4°C and then cleared by centrifugation at 16.000 *g* at 4°C for 20 min. Protein content in the supernatant was measured in triplicate by the bicinchoninic acid method, accepting a 5% coefficient of variance (Pierce Rockford, IL). Then, the lysates were diluted in Milli-Q ultrapure water and 6×laemlii-buffer (340 mM TRIS-base pH 6.8, 11% SDS, 20% glycerol, 0.05% bromophenol blue and 225 mM freshly added DTT) to the same protein concentration.

### Immunoblotting procedures

Total protein and phosphorylation were measured by standard immunoblotting techniques, largely as described previously [20]. For a given measurement, equal volumes and protein amounts of all samples were separated by SDS-PAGE (PROTEAN Tetra, BioRad, USA) using self-cast 15-well minigels (5–10% acrilamide, 5 gels per full sample set) cast from the same solution. Then, regions of interest were identified based on the molecular weight markers, cut from the gels and semidry-transferred (TE-77, Amersham Biosciences, USA) onto the same PVDF-membrane (Immobilon-P, 0.45 µM, Millipore, USA) for subsequent blocking and antibody-probing. If uneven transfer was suspected based on the molecular weight markers or ECL signal obtained, the blots were ponceau-stained to verify this and all samples rerun for the measurement in question. Primary antibodies used were glucose transporter 4 (GLUT4, Pierce, USA, cat#PA5-1065), hexokinase II (HXII, Santa Cruz, USA, cat#28889), Cytochrome c oxidase I (COXI, Invitrogen, USA, cat# 459600), F1 ATPase (Santa Cruz, USA, cat#16689), SOD2 (Millipore, USA, cat#06-984), Catalase (Abcam, USA, cat#52477), TBC1D1 (provided by C. MacKintosh, University of Dundee, UK), TBC1D4 (Upstate, USA, cat#07-741), alpha1, alpha2, beta1 and beta2 AMPK (provided by D. G. Hardie, University of Dundee, UK), gamma3 AMPK (Zymed, USA, cat#52-5717), eukaryotic elongation factor 2 (eEF2) total (Santa Cruz, USA, cat# 13004), extracellular receptor activated protein kinase (ERK) 1/2 total (Cell Signaling Technology, USA, cat#9102), pan-actin (Sigma, USA, cat#A2668), phospho-ERK 1/2 Thr202/Tyr204 (Cell Signaling Technology, cat#9101), phospho-Thr56 eEF2 (Cell Signaling Technology, USA, cat#2331), phospho-Thr172 AMPK (Cell Signaling Technology, USA, cat#2531). Horse-radish peroxidise-conjugated secondary antibodies were from Dako (Glostrup, Denmark). Band intensity was quantified by Kodak imaging software (Kodak 1D version 3.5, USA). Loading was optimised for signal and linearity for all antibodies prior to use. Coomassie-staining after development was performed by submersion of PVDF membranes for ∼10 min in 0.5% Coomassie Blue G-250 in 50% ethanol/10% acetic acid, removal of excess coomassie-stain with distilled water followed by destaining in 50% ethanol/10% acetic acid until bands were clearly visible. The strong band above 150 kDa was quantified. Half and double protein amounts were loaded for one vastus lateralis sample to show that the signal was in the dynamic range.

### Statistics

Statistical evaluation was performed using SigmaPlot 11.0 (Systat Software Inc., USA) by 1-way ANOVA for protein expression data (muscle), two-way ANOVA with repeated measures (muscle×time) for rest vs. exercise, and one-way ANOVA with repeated measures for blood data. When ANOVA revealed significant differences, a *Tukey's post hoc* test was used to correct for multiple comparison. For the global comparison of EMG-normalised data in Figure 3F, the normality-test failed and the data-set was instead analysed using Wilcoxon's signed rank test. The significance level was set at *p*<0.05. All data are expressed as mean ± SEM.
